# Application Perspectives of Nanomedicine in Cancer Treatment

**DOI:** 10.3389/fphar.2022.909526

**Published:** 2022-07-01

**Authors:** Shanshan Hou, Muhammad Hasnat, Ziwei Chen, Yinong Liu, Mirza Muhammad Faran Ashraf Baig, Fuhe Liu, Zelong Chen

**Affiliations:** ^1^ Department of Pharmacy, Zhejiang Pharmaceutical College, Ningbo, China; ^2^ The Affiliated Cancer Hospital of Zhengzhou University & Henan Cancer Hospital, Henan Province Engineering Research Center of Artificial Intelligence and Internet of Things Wise Medical, Zhengzhou, China; ^3^ Institute of Pharmaceutical Sciences, University of Veterinary and Animal Sciences, Lahore, Pakistan; ^4^ Hospital Laboratory of Nangjing Lishui People’s Hospital, Nangjing, China; ^5^ Laboratory of Biomedical Engineering for Novel Bio-functional, and Pharmaceutical Nanomaterials, Prince Philip Dental Hospital, Faculty of Dentistry, The University of Hong Kong, Hong Kong, Hong Kong SAR, China

**Keywords:** nanomedicine, chemotherapy, gene therapy, immunotherapy, cancer

## Abstract

Cancer is a disease that seriously threatens human health. Based on the improvement of traditional treatment methods and the development of new treatment modes, the pattern of cancer treatment is constantly being optimized. Nanomedicine plays an important role in these evolving tumor treatment modalities. In this article, we outline the applications of nanomedicine in three important tumor-related fields: chemotherapy, gene therapy, and immunotherapy. According to the current common problems, such as poor targeting of first-line chemotherapy drugs, easy destruction of nucleic acid drugs, and common immune-related adverse events in immunotherapy, we discuss how nanomedicine can be combined with these treatment modalities, provide typical examples, and summarize the advantages brought by the application of nanomedicine.

## Introduction

In recent years, the incidence and mortality of cancer have been increasing year by year under the influence of many factors such as population aging, work pressure, and environmental changes, etc. According to the 2020 Global Cancer Statistics Report, there were approximately 19.3 million new cancer cases and 10 million deaths that year (Sung et al., 2021). The treatment of cancer has now become an important issue to be solved urgently in the medical field. For the treatment of cancers, chemotherapy is a commonly used and effective drug therapy. However, its shortcomings and limitations in clinical application have also been shown, such as application limitations due to poor water solubility, serious adverse events caused by non-specific distribution, etc. In recent years, with the development of technology and the innovation of treatment concepts, new treatment methods such as gene therapy and immunotherapy are changing the pattern of tumor treatment (Libutti, 2019; Tan et al., 2020). However, the *in vivo* delivery of anti-tumor gene therapy and immunotherapy drugs still faces challenges, such as low tumor cell uptake rate and poor tumor permeability, which seriously affect the therapeutic effect. Nanoparticulate delivery systems (NDSs)is an important way to optimize drug delivery, which can effectively improve the accumulation, penetration and target cell uptake of drugs in tumor tissue and achieve controllable drug release. Replacing traditional drug delivery with NDSs can enhance the efficacy of treatment and reduce the incidence of adverse events, and has shown significant clinical benefits (Collins and Thrasher, 2015; Raza et al., 2019; Riley et al., 2019; Yahya and Alqadhi, 2021; Raza et al., 2022). In this review, we will review the progress of NDSs and the application of nanomedicine in cancer therapy, focusing on the new progress in the application of nanomedicine in chemotherapy, gene therapy and immunotherapy.

## Nanoparticulate Delivery Systems

Relying on the vigorous development of nanotechnology, NDSs have attracted more and more attention of scientists, and also showed great application value and broad development prospects. Nanomaterials refer to particles with a size of less than 100 nm, or with a size of less than 1 μm that can exhibit nanoparticle properties, and whose structural units are generally smaller than the cell volume. Compared with traditional drug delivery systems, NDSs can effectively improve pharmacokinetics and pharmacodynamics due to their specificity in size, material, and shape (Kinnear et al., 2017). Due to the heterogeneity and complexity of tumors, nanomaterials used in cancer therapy are often designed as multifunctional nanoplatforms, which are usually composed of loaded drugs, structural frameworks and functional units. Compared with traditional drugs, NDSs has obvious advantages in tumor treatment: 1) NDSs can effectively deliver drugs with different physicochemical properties, such as: solving the difficult problem of hydrophobic drug delivery, effectively delivering charged nucleic acid drugs and protecting them from nuclease degradation. 2) NDSs can deliver multiple types of drugs simultaneously. 3) NDSs can simultaneously realize tumor diagnosis and treatment. 4) NDSs can improve the targeting of drugs, and both passive targeting based on enhanced permeability and retention effects and active targeting under functional element modification have demonstrated distinct advantages. 5) NDSs can achieve controlled release of drugs. Namely engineered stimuli-responsive nanomaterials enable precise drug delivery and controlled release under the influence of endogenous or exogenous stimuli (Zhu G. et al., 2017; Bhushan et al., 2017; Liu et al., 2017; Cao et al., 2020; Zhu et al., 2020; Gote et al., 2021; Li et al., 2021; Yang et al., 2021; Liu et al., 2022).

## Chemotherapy Nanomedicine

**FIGURE 1:**
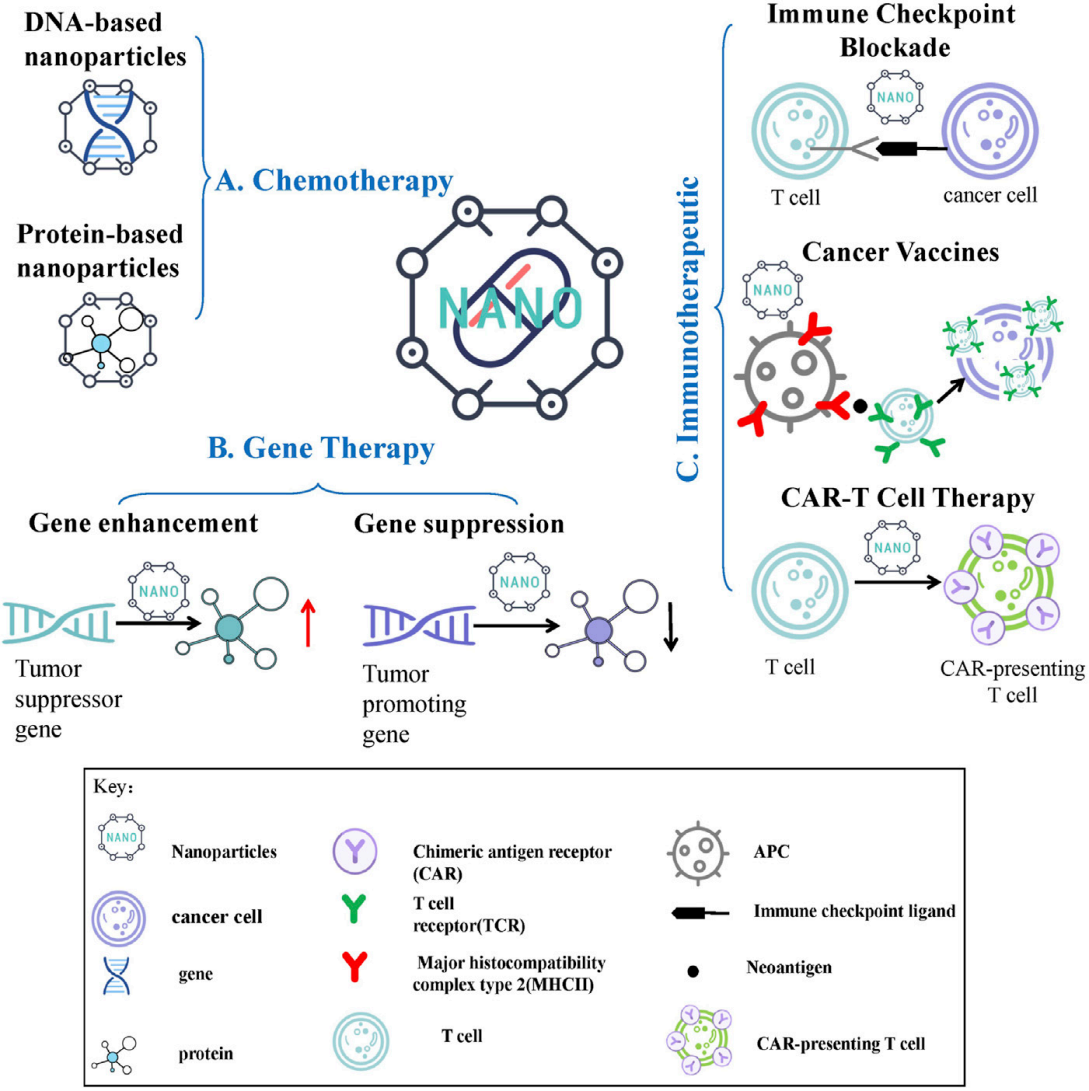
Nanomedicine Delivery Strategies for Chemotherapy, Cancer Gene Therapy and Immunotherapy. **(A)** Chemotherapy nanomedicine: DNA-based nanoparticles and protein-based nanoparticles are shown in this article. **(B)** Gene therapy nanomedicine: gene enhancement therapy and gene suppression therapy are shown in this article. **(C)** Immunotherapeutic nanomedicine: immune checkpoint blockade therapy, cancer vaccines and chimeric antigen receptor T cell therapy are shown in this article.

Chemotherapy is currently one of the most widely used and mature tumor treatment methods in clinical practice. However, there are many problems in the clinical application, such as the lacking target and the large side effects of conventional chemotherapeutic drugs and the poor water solubility of many first-line chemotherapeutic drugs (doxorubicin, paclitaxel, etc.), aggravating the difficulty of clinical treatment. The development and application of nanomaterials optimize the delivery of the above-mentioned chemotherapeutic drugs, and effectively improve the safety and efficacy of chemotherapy (Wei et al., 2021). Nanomaterials currently used for chemotherapeutic drug delivery include organic nanomaterials (Zhang D. Y. et al., 2020), inorganic nanomaterials (Wang C.-S. et al., 2021), composite nanomaterials (Akgöl et al., 2021), and biological nanomaterials (Wang J. et al., 2021). Among them, biological nanomaterials have very high application value in drug delivery because of their high safety, good biocompatibility, easy degradation, and certain targeting properties. Biological nanomaterials include endogenous natural nanomaterials and biomimetic nanomaterials (Navya et al., 2019). Here we take the classic DNA and protein nanomaterials as examples to introduce their application in chemotherapy (Figure 1A).

### DNA-Based Nanoparticles (NPs)

DNA is the carrier of the genetic information of the organism, plays an important regulatory role in the physiological and pathological processes of the organism, and is an ideal natural drug carrier material. As an emerging drug delivery carrier in recent years, DNA-based nanoparticles (NPs) have significant advantages, with excellent degradability, biocompatibility, and sequence programmability (Xu et al., 2021). DNA-based NPs can effectively load a variety of drugs, and achieve targeted drug delivery to tumor tissues with the assistance of specific functional elements, while improving cellular drug uptake and stimuli-responsive drug release. It is an effective tool to solve common problems of chemotherapy (low efficacy of drugs, large toxic and side effects, etc) and many progress have been made in recent years (Lv et al., 2021). In 1982, Seeman’s group proposed that DNA molecules can construct precise and ordered nanostructures based on the A-T, G-C Watson-Crick base pairing principle, which opened the prelude to DNA nanotechnology. Through rational design, DNA molecules can self-assemble into various kinds of 2D or 3D NPs. The existing DNA self-assembly techniques include DNA Tile self-assembly, rolling circle amplification (RCA), DNA origami, and DNA single-stranded tile self-assembly (Lau and Sleiman, 2016; Mohsen and Kool, 2016; Evans and Winfree, 2017; Ji et al., 2021). In physiological environment, self-assembled DNA NPs can resist degradation to a certain extent and show stronger stability than natural single-stranded or double-stranded DNA, which has high clinical application value (Ahn et al., 2020; Ramezani and Dietz, 2020).

At present, DNA-based NPs have successfully achieved effective delivery of chemotherapeutic drugs such as doxorubicin, daunorubicin, platinum, etc (Halley et al., 2016; Zhang L. et al., 2019; Wu et al., 2019). Doxorubicin, used alone or in combination with other drugs, is one of the most effective chemotherapeutic drugs commonly used clinically, which can treat a variety of tumors including solid tumors and hematological tumors by inhibiting DNA synthesis (Carvalho et al., 2009). We take doxorubicin as an example to illustrate how DNA-based NPs deliver chemotherapeutic drugs. At present, DNA-based NPs of various shapes have been reported for the delivery of doxorubicin by virtue of the drug-embedding properties, covering RCA-based nanostructures (Zhao et al., 2018), DNA tetrahedra (Zhang J. et al., 2021), dendritic DNA nanostructures (Li et al., 2020), and nanostructures based on DNA origami technology (Liu J. et al., 2018), in which tubular and triangular DNA nanocarriers based on DNA origami technology have the characteristics of high drug loading (Guan et al., 2021). Interestingly, DNA-based NPs with specific hydrodynamic dimensions and geometries (triangular DNA origami nanostructures) can passively accumulate in tumor tissues, showing excellent targeting (Zhang et al., 2014; Jiang B. et al., 2019). In order to further improve the targeting of chemotherapeutic drugs and reduce the occurrence of adverse reactions, the latest research pays more attention to the progress of modification. With the help of functional elements such as aptamers, DNA-based NPs can improve the efficacy of chemotherapeutic drugs and reduce adverse reactions through active targeting. For example, Zhang et al. demonstrated that Sgc8 aptamer-modified DNA nanoflowers (NFs) can selectively recognize the cell membrane protein tyrosine kinase 7, and Sgc8-NFs-Ferrocene/Doxorubicin can be selectively protein tyrosine kinase 7 positive cancer cells, significantly improving the tumor-targeting ability of the drug (Zhang Q. et al., 2019). In addition, the significantly different physiological characteristics of tumor tissue and normal tissue have inspired scientists to design many tumor microenvironment-responsive NDSs. Compared with normal tissue, tumor tissue has the following different physiological characteristics, including: 1) high concentration of reducing substances in tumor cells; 2) high concentration of ATP in tumor cells; 3) abnormally expressed enzymes in tumor cells, such as telomerase, matrix metalloproteinase, etc; 4) pH imbalance inside and outside tumor cells (Dai et al., 2017; Jin and Jin, 2020). Given this, the designed DNA-based NPs trigger structural reorganization, exposing the coated drug under the influence of external stimuli such as pH (Zhao et al., 2018), reducing environment (Liu X. et al., 2018), enzymes (Zhang G. et al., 2017), ATP (Lu et al., 2018), etc., which better achieves the precise delivery and release of the drug, thereby effectively increasing the anti-tumor effect of the chemotherapeutic drugs and reducing the damage to normal tissues. For example, Zhao et al. (2018) developed a doxorubicin-loaded DNA NPs with a cancer cell targeting Sgc8 aptamer and a hairpin structure showing pH-responsive doxorubicin loading-releasing capability, which exhibited good biological stability, comparable to doxorubicin loading capacity, specific tumor targeting capacity, and sustained release of pH responsive doxorubicin. The delivery of doxorubicin with the designed DNA-based NPs realized the targeted, controllable and precise release of chemotherapeutic drugs, which enhanced the efficacy of the drug and reduced the occurrence of adverse events. The applications of other DNA nanoparticles in chemotherapy are shown in Table 1.

**TABLE1:** DNA nanoparticles in chemotherapy.

Chemotherapeutic Drugs	DNA Nanostructures	Modification	Effect	Ref
Doxorubicin	DNA tetrahedron	Folate receptor	Apoptosis promoting	Zhang et al. (2017b)
		KLA peptide	Drug delivery and apoptosis promoting	Yan et al. (2020)
		AS1411 + MUC1 aptamer	Breast cancer cell imaging and drug delivery	Liu et al. (2018a)
		Affibody	Selectivity and inhibition of breast cancer cells	Zhang et al. (2017c)
	DNA octahedron	Folate	Selective targeting	Raniolo et al. (2018)
	DNA icosahedron	MUC1 aptamer	Efficient and specific internalization for killing epithelial cancer cells	Chang et al. (2011)
	DNA NFs	Sgc8	Nuclease resistance and binding of different functional moieties	Lv et al. (2015)
	DNA triangle and tube	-	Increased doxorubicin cellular internalization and elevated susceptibility to drug-resistant adenocarcinoma cells	Bertrand et al. (2014)
	RCA-based nanostructures	Imotif sequence, Sgc8	pH-Responsive Drug Delivery	Zhao et al. (2018)
Daunorubicin	DNA nanorod	-	Circumvent drug-resistance mechanisms in a leukemia model	Halley et al. (2016)
Platinum	DNA tetrahedron	-	Targeted platinum drug delivery	Wu et al. (2019)
	DNA icosahedron	Telomerase-Responsive	Precise delivery of platinum nanodrugs to cisplatin-resistant cancer	Ma et al. (2018)

### Protein-Based Nanoparticles (NPs)

Also as a very important natural biological macromolecules, proteins have been widely constructed as nanocarriers for chemotherapeutic drugs in recent years. Similar to DNA-based NPs, protein-based NPs also have many outstanding advantages, such as: 1) good biocompatibility and degradability; 2) natural biological sources, high availability and low toxicity; 3) unique three-dimensional structure; 4) amphiphilic of hydrophilic and hydrophobic molecules or solvents; 5) amino, carboxyl and hydroxyl groups for chemical bonding (Martínez-López et al., 2020). Proteins often designed as nanocarriers mainly include albumin, transferrin, ferritin, low-density lipoprotein, high-density lipoprotein, etc (Iqbal et al., 2021). The application of protein-based NPs to deliver chemotherapeutic drugs has been widely studied, and has shown good clinical application prospects.

When it comes to protein-based NDSs, the most classic one belongs to albumin-bound paclitaxel. Paclitaxel, a hydrophobic anticancer drug, needs to be dissolved in polyoxyethylene castor oil for clinical use, which may cause severe allergic reactions in some patients. Paclitaxel was loaded into albumin via hydrophobic interaction and then constructed into albumin-bound paclitaxel NPs with a diameter of 130 nm, which is traded as Abraxane (Celgene) (Zhang Y. et al., 2020). As the most abundant serum protein in the human body, albumin does not have the immune response caused by castor oil, which not only solves the problem of allergies, but also improves the efficacy and reduces the toxicity to normal tissues and organs. Currently, Abraxane has become one of the best-selling anticancer drugs and is a milestone in protein-based NPs (Yardley, 2013). In addition to non-covalent interactions such as hydrophobic interactions and electrostatic interactions, albumin has abundant binding sites (amine, thiol and carboxyl groups) and also can be used in combination with chemotherapeutic drugs, showing the huge application prospects of albumin (Hoogenboezem and Duvall, 2018; An and Zhang, 2017). Multiple clinical trials of Albumin NPs are currently being pursued, which we list in Table 2. Such as NCT01673438, NCT01580397, NCT02014844, NCT02049905, NCT00477529, NCT00635284, NCT02009332, NCT02494570 (https://clinicaltrials.gov/). Moreover, with the deepening of the research on protein-based NPs, stimuli-responsive NDSs have emerged to further improve the targeting efficiency, which can specifically respond to stimuli in the tumor microenvironment and precisely deliver the drug to the tumor area, enabling targeted therapy (Mi, 2020). As an example, Yang et al. (2020) designed and developed a doxorubicin-loaded NPs containing pH-sensitive Schiff bovine serum albumin, which not only has a high loading capacity but can also trigger the release of doxorubicin by pH.

**TABLE 2:** Albumin nanoparticles approved or in clinical trials for chemotherapy.

Chemotherapeutic Drugs	Name	Indication(s)	Clinicaltrials Gov Identifier
Paclitaxel	Nab-paclitaxel (Abraxane)	non-small-cell lung cancer; Breast cancer; pancreatic cancer	Approved
Doxorubicin	Al-doxorubicin (DOXO-EMCH INNO-206)	Advanced solid tumour	NCT01673438
		Pancreatic ductal adenocarcinoma	NCT01580397
		Glioblastoma	NCT02014844
		Metastatic, locally advanced or unresectable soft tissue sarcoma	NCT02049905
Docetaxel	Nab-docetaxel (ABI-008)	Hormone-refractory prostate cance	NCT00477529
Rapamycin	Nab-rapamycin (ABI-009)	Solid tumours	NCT00635284
		Non-muscle-invasive bladder cancer	NCT02009332
		PEComa	NCT02494570

Another class of proteins commonly constructed as NDSs are iron homeostasis-related proteins, including transferrin and ferritin, both of which have excellent performance in receptor-mediated active targeted delivery. Transferrin, mainly produced by hepatocytes in the human body, is the main iron-containing hydrophilic transporter in plasma, and is mainly responsible for transporting iron in hepatocytes to cells in other tissues. Transferrin is composed of a single-chain glycoprotein and is divided into two evolutionary lobes, namely C-lobe (343 amino acids) and Nlobe (336 amino acids), which are connected to each other by a short spacer (Elsayed et al., 2016; Gou et al., 2018; Zhang et al., 2018). Transferrin mediates iron uptake by binding to the transferrin receptor (TfR). TfR, a transmembrane glycoprotein including TfR1 and TfR2, is the major protein receptor for iron metabolism *in vivo* (Fernandes et al., 2021). TfR is expressed in both normal and cancer tissues. However, studies have confirmed that the expression rate of TfR in cancer cells is nearly 100 times higher than that in normal cells. Cancer cells grow rapidly and their demand for iron is greatly increased during DNA synthesis, differentiation and regeneration. Therefore, TfR expression is increased in cancer cells in order to adapt to the increased iron requirement and maintain rapid cell division (Candelaria et al., 2021; Guo et al., 2021).

Based on this active transport capability, transferrin NPs can be used for tumor diagnosis and targeted delivery of therapeutic drugs. Goswami et al. designed Transferrin -templated copper nanoclusters-doxorubicin NPs for bioimaging and targeted drug delivery (Goswami et al., 2018). In addition, transferrin can also be used alone as a ligand to provide active tumor targeting capabilities of drug delivery systems. Research by Wei et al.showed that doxorubicin -loaded Transferrin-binding peptide CGGGHKYLRW significantly enhanced the antitumor efficacy in mice bearing HCT-116 tumors compared to polymersomes without transferrin binding (Wei et al., 2020).

Ferritin, found in most living cells, is the major iron storage protein in organisms. At present, various sources of ferritin have been used in biological nanocarriers, including human ferritin (HFt), horse spleen ferritin (HoSF), *Archaeoglobus fulgidus* ferritin (AfFtn), *Pyrococcus furiosus* ferritin (PfFt), and rat heavy-chain ferritin (Zhang P. et al., 2021; Zafar et al., 2021). HFt is a naturally formed spherical hollow nanocages with an outer diameter of 12 nm and an inner diameter of 8 nm, which is composed of 24 subunits of two types: heavy chain ferritin (HFn) and light chain ferritin (LFn). The inner cavity of each ferritin can store up to 4,500 Fe^3+^ atoms (Chakraborti and Chakrabarti, 2019). At the junction of these subunits, there are 8 hydrophilic channels and 6 hydrophobic channels, wherein the hydrophobic channel has three-fold symmetry and the hydrophilic channel has four-fold symmetry. In particular, hydrophilic channels are flexible enough to allow larger-sized molecules to penetrate into the ferritin cavity (Zhao et al., 2016; Tesarova et al., 2020). More importantly, HFn can selectively bind to tumor cells *via* TfR1 mediated specific targeting followed by rapid internalization *in vitro* and *in vivo*. Therefore, HFn can selectively deliver therapeutic drugs into tumors (Zang et al., 2017; Cheng et al., 2020). The unique nanocage structure and inherent tumor-targeting properties of ferritin make it a highly valuable biological nanocarrier. However, despite the above advantages, the low purification efficiency of natural protein brings difficulties to its practical application, and the recombinant ferritin constructed in various ways makes up for the above shortcomings (Veroniaina et al., 2021). According to the characteristics of ferritin nanocages, metal-based chemotherapeutic drugs such as carboplatin and cisplatin are the first to achieve effective precipitation in the cavity of iron nanocages. Subsequently, ferritin nanocages have also been used to load other non-metallic chemotherapeutic drugs such as doxorubicin (Song et al., 2021). Here we list the applications of ferritin in the delivery of chemotherapeutic drugs in Table 3.

**TABLE 3:** Ferritin nanoparticles in chemotherapy.

Chemotherapeutic Drugs	Ferriatin Types	Indication(s)	Ref
Doxorubicin	human HFn	Targeting drug delivery	(Liang et al., 2014; Gu et al., 2020; Inoue et al., 2021)
	Recombinant human HFn	Targeting drug delivery	Zhen et al. (2013)
	Recombinant HFt	Targeting drug delivery	Falvo et al. (2018)
	HoSF	Targeting drug delivery	(Kilic et al., 2012; Zhang et al., 2019a)
	PfFt	Hepatocellular carcinoma Targeting drug delivery	Jiang et al. (2019b)
Cisplatin	Recombinant human HFn	Targeting drug delivery	(Falvo et al., 2013; Monti et al., 2019)
	HoSF	Targeting drug delivery	Xing et al. (2009)
Oxaliplatin	Recombinant human HFn	Targeting drug delivery and photodynamic therapy	Liu et al. (2020b)
	HoSF	Targeting drug delivery	Xing et al. (2009)
Paclitaxel	Recombinant human HFn	Targeting drug delivery glioma	Liu et al. (2020c)
Epirubicin	HoSF	Targeting drug delivery	Tan et al. (2018)
	Recombinant human HFn	Targeting drug delivery	Wang et al. (2022)
Mitoxantrone	Recombinant human HFn	Targeting drug delivery Tumor therapy (colon, breast, sarcoma and pancreas)	Falvo et al. (2018)

Lipoproteins are mainly responsible for the transport of cholesterol and various lipids in human blood vessels. According to their different densities, human plasma lipoproteins can be divided into chylomicrons, very low density lipoproteins (VLDL), and low density lipoproteins (LDL). and high-density lipoprotein (HDL) (Salter and Brindley, 1988). Lipoproteins are spherical biological macromolecules. Each lipoprotein molecule consists of a non-polar or hydrophobic core composed of cholesterol or triglycerides, and an outer shell of phospholipids and apoproteins, whose structure is a natural NPs (Orlova et al., 1999). Based on structural features and size advantages (less than 25 nm), LDL and HDL are commonly used to deliver chemotherapeutic drug.

LDL is a kind of spherical particles with a diameter of 19–25 nm. Its core is 1,500 cholesteryl esters wrapped by an outer layer of 800 phospholipids and 500 unesterified cholesterol molecules. The hydrophilic head of the phospholipid molecules is exposed outside, making the LDL is soluble in the blood (Hevonoja et al., 2000; Sherman et al., 2003). LDL is a very potential delivery vehicle, which has good biocompatibility as a biological macromolecule. Another very important reason is that there is apolipoprotein B-100 (ApoB-100) with a relative molecular weight of 514 kDa in the outermost layer of LDL, which can be recognized by and bind to LDL receptors with high specificity. Moreover, studies have confirmed that LDL receptors are highly expressed on the surface of tumor cells, which provide the necessary lipid matrix for the synthesis of membrane systems for fast growing tumor cells (Ng et al., 2011; Harisa and Alanazi, 2014). As a delivery carrier, LDL can encapsulate various hydrophobic loads such as photosensitizers, radiolabels, and various chemotherapeutic drugs (Thaxton et al., 2016; Li et al., 2019; Di and Maiseyeu, 2021). Lo et al. (2002) conjugated doxorubicin to the ApoB protein of LDL, and the results showed that it significantly reduced the occurrence of adverse reactions. Meanwhile, LDL can also take advantage of the inherent targeting ability of LDL through the strategy of combining with independent nanocarriers. Such as Zhu et al. reported a new “binary polymer” low-density lipoprotein-N-succinyl chitosan-cystamine-urocanic acid with dual pH/redox sensitivity and targeting effect, which was synthesized for the co-delivery of breast cancer resistance protein small interfering RNA and paclitaxel, showing significant tumor targeting and effectively inhibited tumor growth (Zhu WJ. et al., 2017). In addition, natural LDL is derived from plasma separation, which is difficult to produce on a large scale, and there are more and more studies on recombinant/synthetic LDL (Zhu G. et al., 2017). For example, Li et al. (2019) constructed a pH-sensitive ApoB-100/oleic acid-doxorubicin/nanostructured lipid carrier NPs, similar to LDL, which showed a increased accumulation at tumor site, pH-dependent release of doxorubicin, and potent breast cancer inhibition.

HDL is an endogenous nanocarrier with a structure similar to LDL and a diameter of 8–13 nm (Mulder et al., 2018). Similarly, blood HDL cholesterol levels in cancer patients are also lower than in normal healthy people (Ganjali et al., 2021). Unlike LDL, which recognizes receptors and mediates endocytosis, HDL specifically recognizes scavenger receptor class B type 1 (SR-B1) and the main protein of HDL stays on the surface of the cell membrane, while its core lipophilic cholesteryl ester directly enters the cell cytoplasm (Krieger, 1999). Furthermore, the major apolipoproteins of HDL contain fewer amino acids than those of LDL, effectively avoiding the formation of large irreversible aggregates (Mulder et al., 2018). The above-mentioned points all provide a good basis and favorable conditions for HDL as a delivery vehicle. It is rare to use natural HDL as a delivery vehicle in existing reports, and recombinant HDL (rHDL) NPs can be used to deliver chemotherapeutic drugs. For example, Wang et al. constructed a doxorubicin -loaded rHDL NPs with high affinity for SR-B1 to treat liver cancer. The results confirm that doxorubicin can be efficiently delivered into cells. After SR-B1 was blocked with antibodies, the delivery efficiency was significantly reduced, which well confirmed that the drug delivery efficiency was dependent on the active targeting of SR-B1 expressed in tumors by apolipoprotein A-1 contained in rHDL NPs (Wang et al., 2014). In addition, rHDL can also be used as a co-delivery carrier for the combination therapy of multiple chemotherapeutic drugs, or the combination therapy of chemotherapy and immunotherapy (Iqbal et al., 2021; Mei et al., 2021). Such as Rui et al. (2017) developed a developed a rHDL NPs for paclitaxel and doxorubicin, which were remarkably effective in increasing the ratiometric accumulation of drugs in cancer cells and enhancing antitumor response at synergistic drug ratios. In particular, they exhibited more efficacious anticancer effects in an *in vitro* cytotoxicity evaluation and in a xenograft tumor model of hepatoma compared with free drug cocktail solutions. Scheetz et al. (2020) co-loaded docetaxel and cholesterol-modified Toll-like receptor 9 agonist CpG oligonucleotides in synthetic HDL to prepare a nanoplatform for combined chemotherapy and immunotherapy. Compared with chemotherapy alone, it can be used for significant Improve survival outcomes.

## Gene Therapy Nanomedicine

In recent years, with the increasing maturity of gene manipulation technologies such as gene silencing and gene editing, scientists have begun to treat various diseases by site-specific up-regulation or down-regulation of target genes, and have achieved certain progress and widespread attention, especially in cancer therapy. The drugs used in gene therapy are nucleic acid therapeutics with lower cytotoxicity, which show significantly fewer adverse reactions and better therapeutic effects compared with conventional treatments such as chemotherapy (Gutierrez et al., 1992). However, gene therapy also faces certain difficulties. For example, commonly used gene therapy agents are not easily taken up by cells and have poor stability during circulation *in vivo*; traditional viral vectors (lentivirus, adenovirus, adeno-associated virus, etc.) are limited in application due to safety concerns such as insertional mutagenesis and immunogenicity. NDSs solve the above problems well, showing low toxicity and immunogenicity, high payload capacity, sustained and controlled release characteristics. Commonly used gene therapy strategies include gene enhancement therapy and gene suppression therapy, in which the application value of nanomedicine will be described here (Figure 1B) (Rui et al., 2019).

### Gene Enhancement Therapy

Gene enhancement therapy generally refers to “expressing a certain gene” or “expressing a certain protein” by introducing a plasmid or mRNA. Tumor suppressor genes can inhibit cell proliferation when activated or overexpressed. Overexpression of one or more tumor suppressor genes can effectively inhibit the growth and progression of tumors, among which protein 53 (*p53*)gene and phosphatase and tensin homolog (*PTEN*)gene are the most classical and the most deeply studied (Lee and Muller, 2010; Álvarez-Garcia et al., 2019; Lacroix et al., 2020). Both mRNA and plasmid can achieve protein expression, but the way via mRNA quickly and without mutation, integration or other adverse events, which is safer and more efficient (Akeno et al., 2015; Que et al., 2018; Sobhani et al., 2021). However, RNA molecules are unstable and impermeable to membranes, requiring rational design of delivery systems. Kong et al. designed a redox-responsive NPs platform for efficient delivery of mRNA encoding *p53*, which delayed the growth of hepatoma and non-small cell lung cancer cells by inducing cell cycle arrest and apoptosis (Kong et al., 2019). Islam et al. reintroduced *PTEN* mRNA into *PTEN*-null prostate cancer cells through polymer-lipid hybrid NPs coated with polyethylene glycol shell, which significantly inhibited tumor growth (Islam et al., 2018). In addition, the suicide gene therapy systems are also commonly used for gene enhancement, such as herpes simplex virus thymidine kinase (HSV-TK), of which TK gene is a drug-susceptibility gene. After tumor cells were transfected with this gene, they were sensitized and killed by the nontoxic prodrugs glycoxyguanosine or acyclovir (Zhao et al., 2014). A study reported that *in vivo* delivery of the TK-p53- nitroreductase triple therapeutic gene by poly (D,L-lactic-co-glycolic acid)-poly (ethylene glycol)-Polyethylenimine NPs functionalized with SP94 peptide (a peptide that targets hepatocytes) restored *p53* function and enhanced cancer cells’ response to the prodrug ganximation glycoxyguanosine and CB 1954 (Sukumar et al., 2020). Due to the negative charge of mRNA, most of the NPs currently used to deliver mRNA drugs contain a cationic gradient, which can form stable complexes with mRNA to achieve high loading rates, such as ionizable lipid NPs (Ding et al., 2021), polymer-lipid hybrids NPs (Islam et al., 2018), and biological nanostructures with higher biocompatibility (Li et al., 2017; Forterre et al., 2020), etc.

### Gene Suppression Therapy

Gene suppression can also treat cancer by silencing specific genes that produce abnormal or harmful proteins, such as small interfering RNA (siRNA) therapy. Several *in vitro* and *in vivo* studies have confirmed that siRNA-mediated silencing can significantly inhibit abnormal cancer cell proliferation (Shi et al., 2019; Han et al., 2021; Krishn et al., 2022). In addition, siRNA can sensitize drug-resistant cancer cells, showing great promise in enhancing chemotherapy (Shen et al., 2020). An ideal delivery system should protect siRNA from degradation by nucleases, as well as deliver and release it into the cytoplasm of targeted tumor cells without adverse effects. At present, the research on nanocarriers for siRNA delivery has been relatively mature, including lipid nanocarriers, polymer NPs, dendrimers, inorganic NPs, etc (Babu et al., 2017; Subhan and Torchilin, 2019). In addition, the aforementioned biological NPs can also deliver siRNA with high loading and high biocompatibility. Wang et al. constructed a DNA nanodevice using DNA origami technology to co-deliver siRNA and the doxorubicin (Nanodevice-siBcl2-si P-glycoprotein- doxorubicin), which induced potent cytotoxicity and tumor growth inhibition with no observable systemic toxicity (Wang X. et al., 2021).

CRISPR/Cas9 gene editing technology, another cancer gene-suppression therapy, has the potential to permanently destroy tumor survival genes, which overcomes the repeated dosing limitations of traditional cancer therapy and improves the therapeutic effect (Rafii et al., 2022). CRISPR/Cas9 consists of two parts: Cas9, a nuclease that cuts DNA at a target site, and a single guide RNA (sgRNA) that directs Cas9 to cut at a specific or desired site in DNA. Since the CRISPR/Cas9 complex requires manipulation of the nuclear genome, its components need to be translocated into the nucleus (Zhan et al., 2019; Zhang S. et al., 2021). Therefore, it is necessary to overcome the barriers of tissue and cell membranes to effectively deliver CRISPR/Cas9 to the target site, facing considerable challenges. In recent years, nanomaterials have gradually shown unique advantages in gene delivery. Currently developed CRISPR/Cas9 NDSs include cationic liposomes (Yin et al., 2020), lipid NPs (Rosenblum et al., 2020), cationic polymers (Zhang Y. et al., 2019), vesicles (Horodecka and Düchler, 2021), and gold NPs (Tao et al., 2021). In order to better reduce the off-target effects, researchers have developed a stimulus-based intelligent NDSs. Intelligent NPs can be based on endogenous signals (including pH, redox and ATP) and exogenous signals (including radiation, magnetic ultrasound), to control or regulate the delivery of CRISPR/Cas9 to specific cells. For example, Wang et al. designed a multifunctional NPs modified with pH-sensitive epidermal growth factor receptor targeting and nuclear guide peptides to efficiently deliver CRISPR/Cas9 and epirubicin to the human tongue squamous cell carcinoma SAS cells and SAS tumor mice, providing a pH-responsive co-delivery platform for chemotherapy and CRISPR/Cas (Wang Z. et al., 2021). Another study reported near-infrared light-responsive nanocarriers of CRISPR-Cas9 to inhibit tumor cell proliferation *in vitro* and *in vivo* through near-infrared light-activated gene editing (Pan et al., 2019). DNA-based NPs can load Cas9/sgRNA complexes by sequence hybridization. Shi et al. designed a miR-21 (overexpressed in tumor cell)-responsive Cas9/sgRNA ribonucleoprotein delivery system based on DNA NFs, which could significantly improve genome editing efficiency and make it possible to control the expression of endogenous genes in a cell-type-specific manner through specific endogenous or exogenous miRNAs (Shi et al., 2020).

## Immunotherapeutic Nanomedicine

In recent years, immunotherapy has developed rapidly and gradually matured, and its emergence has revolutionized the treatment standard and treatment concept of cancer (Abdelbaky et al., 2021; Aktar et al., 2022). Radiotherapy and chemotherapy of traditional treatment methods generally use toxic drugs or radiation to directly ablate cancer cells. However, the target of tumor immunotherapy is mainly immune cells, which can activate the body’s anti-tumor immune response to kill tumor cells by inhibiting negative immune regulators and enhancing the ability of immune cells to recognize tumor cell surface antigens (van den Bulk et al., 2018; Zhong et al., 2020). In the early stage of tumor immunotherapy, the main method is to directly attack tumor cells with cytokines produced by immune cells. With the gradual deepening of cancer immunotherapy research, immune checkpoint inhibitors, tumor vaccine immunization and chimeric antigen receptor T (CAR-T) cell therapy have emerged, and they have become the main force in immunotherapy (Sahin and Türeci, 2018; Ma et al., 2019; Galluzzi et al., 2020; Holstein and Lunning, 2020; Raza et al., 2021) With the deepening of research, the value of nanomedicine in tumor immunotherapy is constantly emerging (Figure 1C).

### Immune Checkpoint Blockade Therapy

Inhibitory immune checkpoints can suppress the body’s immune response and prevent the occurrence of autoimmunity. Tumor cells can suppress the body’s immune response, thereby evading clearance by the body’s immune system via expressing inhibitory immune checkpoint molecules that interact with T cells (Jhunjhunwala et al., 2021). Currently widely studied immune checkpoints are cytotoxic-T-lymphocyte-associated protein 4 (CTLA-4), program death 1 (PD-1) and program death-ligand 1 (PD-L1), according to which monoclonal antibodies (mAbs) drugs are designed such as ipilimumab (CTLA-4 inhibitor), pembrolizumab (PD-1 inhibitor), atezolizumab (PD-L1 inhibitors) have been approved by the FDA for marketing (Vaddepally et al., 2020). Nanomedicine is being adapted in various ways to improve immune checkpoint inhibitors (ICIs), increasing the effectiveness and surpassing the limitations. MAbs are difficult to penetrate the blood-brain barrier, and NPs -mediated ICIs mAbs are an effective way to solve this problem (Diesendruck and Benhar, 2017). Galstyan et al. covalently attached ICIs (anti-CTLA-4 and/or anti-PD-1) to poly-β-l-malic acid biopolymer scaffolds, and such nanoscale immunoconjugates allow ICIs mAbs to cross the blood-brain barrier to the tumor environment and modulate immune responses. In particular, the use of nanoscale immunoconjugates has shown promising antitumor activity in the treatment of glioblastoma (Galstyan et al., 2019). Moreover, NDSs have also shown their advantages in reducing the dosage of ICIs or controlling immune-related adverse events (irAIEs) Meir et al., reported that αPDL1-conjugated gold NPs effectively prevented tumor growth at a dose reduced to 1/5 the clinical standard of care dose (Meir et al., 2017). Shen et al. coated PD-L1-overexpressing mesenchymal stem cells plasma membranes on polylactic-co-glycolic acid NPs to design immunosuppressive NPs, managing and reducing irAIEs (Shen et al., 2021). Furthermore, nanocarriers can also be designed as smart platforms for controlled drug release in response to different stimuli present in the tumor microenvironment, which is expected to further enhance the therapeutic efficacy of nanoformulations. For example, researchers have developed a class of liposomes that are dual-responsive to pH and matrix metalloproteinases in combination with PD-L1 inhibitor conjugates and low-dose chemotherapy doxorubicin. In an *in vivo* mouse B16F10 melanoma model, the synergistic effect of chemotherapeutic agents and ICIs enabled dual-responsive liposomes to achieve an optimal tumor suppression efficiency of 78.7% (Liu et al., 2019). Besides, ICIs do not elicit adequate responses in the vast majority of patients with poorly immunogenic tumors due to targeting only major inhibitory axes. Therefore, combining ICIs with nanotechnology-driven immunostimulatory treatments (e.g., nanochemicals, light, and thermal therapy) may help to locally break immune tolerance and enhance systemic antitumor immunity, thereby expanding the availability of proportion of cancer patients benefiting from treatment. Based on the above, the role of nanomaterials in immunotherapy has gone beyond the concept of adjuvant or carrier, which is an effective means to improve the efficacy of ICIs and reduce their toxicity through rational design. At the same time, the dosing cycle and interval time, possible off-target potential and other aspects should be improved to obtain the best solution (Cremolini et al., 2021).

### Cancer Vaccines

Cancer vaccines kill tumor cells without damaging healthy cells by activating the body’s immune system, and they can trigger immune memory to provide long-term protection against tumor recurrence. As a potential drug development concept, cancer vaccines are extremely valuable whether they are used alone or in combination with other immunotherapies (Igarashi and Sasada, 2020; Saxena et al., 2021). With the deepening of research, the advantages of applying nanomedicine in cancer vaccines have gradually emerged (Liu J. et al., 2020). Cancer vaccines are typically combinations of immunogenic components (eg, neoantigens and adjuvants) that are delivered to antigen-presenting cells in peripheral lymphoid tissue. First, encapsulating immunogenic components in nanocarriers can prevent antigen degradation and effectively improve antigen stability (Zhang Z. et al., 2019). Second, nanovaccines co-encapsulate and co-deliver antigens and adjuvants, which can effectively enhance the immunogenicity and therapeutic efficacy of vaccines (Zhu WJ. et al., 2017). Heo and Lim et al. developed a poly (lactic-co-glycolic acid) NPs loaded with ovalbumin for the activation of DCs through the toll-like receptor 7, which proved that the nanovaccine loaded with multivalent antigens and adjuvants can effectively reduce tumor volume (Heo and Lim, 2014). Further, nanovaccine can achieve efficient delivery to immune organs (lymph nodes, spleen). Through rational design of physical properties (such as size, colloidal stability, electrostatic interactions, deformability) or chemical properties (such as light, pH, and enzyme responsiveness), nanovaccines are able to deliver more antigens from injection sites or tumors to lymph nodes, or delivered from the blood to the spleen (Evans et al., 2018; Musetti and Huang, 2018; Chen et al., 2020; Gupta et al., 2021). In particular, nanovaccines further modified by targeting ligands can also be actively targeted and delivered to specific subregions of immune cells (Cai et al., 2021). For example, a click chemistry-based active lymphatic accumulation system was developed to enhance the delivery of antigens and adjuvants to the lymphatic subcapsular sinus (Qin et al., 2021). Ultimately, NPs can enhance immune responses through sustained or controlled release capabilities. For example, Chen et al. (2018) showed that a single injection of clay NPs sustained the release of immunogenic agents, which significantly enhanced the immune response in regional lymph nodes for up to 35 days.

### CAR-T Cell Therapy

One of the strategies for immune evasion of tumor cells is to reduce the expression of their surface antigens, so that T cells cannot be activated in a human leukocyte antigen-dependent manner, thereby evading the attack of the immune system (Pham et al., 2018). CAR-T works by modifying a patient’s own T cells to more effectively recognize and kill tumor cells (Huang et al., 2020). Initially, the application of nanomedicine to CAR-T therapy was mainly to replace viral vectors for *in vitro* genetic modification of T cells to reduce costs and improve safety (Olden et al., 2018; Billingsley et al., 2020). However, *in vitro* CAR-T cell programming is complicated and expensive, and one solution is to program T cells *in vivo*. Nanomedicines were shown to directly construct chimeric antigen receptors *in situ* on circulating T cells without *ex vivo* manipulation in mouse models. Smith et al. designed a polymer NPs carrying a chimeric antigen receptor (CAR)-encoding plasmid and injected leukemia-targeting CAR genes can be efficiently introduced into T cell nuclei, followed by efficient leukemia regression in mice, which is comparable to *ex vivo* programmed CAR-T cells (Smith et al., 2017). Subsequently, this team reported an injectable nanocarrier that deliverd *in vitro* transcribed CAR or T cell receptors mRNA for transient reprogramming of circulating T cells to recognize disease-associated antigens (Parayath et al., 2020). Another important advantage of nanomedicine in this field is the safe and effective enhancement of T cell therapy. Tang et al. (2018) designed a T cell receptors signaling-responsive protein nanogel to co-deposit immunostimulatory cytokines, such as interleukin-15 agonists, onto the surface of CAR-T cells, which significantly extended the therapeutic window and improved tumor clearance in CAR-T cell therapy against solid tumors.

## Perspectives and Future Directions

In recent years, the concept, method and pattern of tumor treatment are constantly changing, which provides a broad space and prospect for the application of nanomedicine. It is the application of intelligent NDSs for tumor chemotherapy, gene therapy and immunotherapy to solve the problem of drug (chemotherapy, biological drug) delivery, optimize its delivery efficiency, and achieve targeted, precise and controllable delivery to a certain degree. However, how to translate preclinically studied antitumor nanomedicines into clinically feasible therapeutics still faces several key challenges. For example: 1) how to optimize patient population stratification in clinical trials; 2) how to optimize the dosing regimen of nanomedicines in combination therapy; 3) how to ensure high quality and reproducibility for industrialized production of nanomedicines, etc. Expectantly, with the deepening of nanotechnology research, the combination of molecular-level scientific design and precise control of process engineering is expected to overcome the core technology of NDSs research and development, thereby opening a new situation for NDSs.

## Glossary

NDSs,: nanoparticulate delivery systems
NPs,: nanoparticles
NFs,: nanoflowers
RCA,: rolling circle amplification
TfR,: transferrin receptor
HFt,: human ferritin
HoSF,: horse spleen ferritin
AfFtn,: Archaeoglobus fulgidus ferritin
PfFt,: Pyrococcus furiosus ferritin
HFn,: heavy chain ferritin
LFn,: light chain ferritin
VLDL,: very low density lipoproteins
LDL,: low density lipoproteins
HDL,: high-density lipoprotein
rHDL,: recombinant HDL apolipoprotein B-100
SR-B1,: scavenger receptor class B type 1
p53,: protein 53
PTEN,: phosphatase and tensin homolog
HSV-TK,: herpes simplex virus thymidine kinase
mRNA,: messenger RNA
siRNA,: small interfering RNA
sgRNA,: single guide RNA
CAR-T,: chimeric antigen receptor T
CTLA-4,: cytotoxic-T-lymphocyte-associated protein 4
PD-1,: program death 1
PD-L1,: program death-ligand 1
CRISPR,: clustered regularly interspaced short palindromic repeat
mAbs,: monoclonal antibodies
ICIs,: immune checkpoint inhibitors
irAIEs,: immune-related adverse events
